# Editorial: The pernicious relationship between social determinants and mental health disorders

**DOI:** 10.3389/fpsyt.2023.1252739

**Published:** 2023-08-08

**Authors:** Ingrid Vargas-Huicochea, Shoshana Berenzon

**Affiliations:** ^1^Departamento de Psiquiatría y Salud Mental, Facultad de Medicina, Universidad Nacional Autónoma de México, Mexico City, Mexico; ^2^Dirección de Investigaciones Epidemiológicas y Psicosociales, Intituto Nacional de Psiquiatría “Ramón de la Fuente Muñiz”, Mexico City, Mexico

**Keywords:** social determinants of health, mental health, depression, psychosis, health workers, family, sociocultural environment

Social determinants of health are those socioeconomic, political, and environmental circumstances that affect people's health, including the conditions in which people are born, live, work, and age, as well as access to health systems.

Differences in social determinants lead to health inequities, which can be modified, and even reduced, by means of specific policies and programs (1). These health inequities occur both between and within countries and generally follow a social gradient, occurring along a continuum and affecting all members of the population, not just the poorest or most disadvantaged (2).

In the domain of mental health, the social gradient affects both the risk of experiencing a mental disorder and access to services, consequently also affecting the prognosis of the person suffering from such a disorder (2–5). It is pertinent to note that there is a bidirectional relationship between mental health disorders and social determinants, as poor mental health can aggravate personal choices and affect living conditions in such a way as to limit an individual's opportunities (6).

The accumulating evidence regarding the relationship between social determinants and mental disorders supports the need for implementation of multilevel interventions to eliminate systemic social inequalities, such as access to educational and employment opportunities, healthy food, and safe housing (6). Similarly, actions aiming to improve the family and work lives of people with mental illness have also been shown to enhance community functioning, perceived wellbeing, and self-esteem. Community actions focusing on decreasing the effects of violence and crime (7) are examples of successful interventions that can decrease mental health inequalities.

Because the vast body of information generated means that we can only analyze some of the many complex links involved in the relationship between social determinants and mental health, it is essential to continue studying the differential effects of these determinants in specific populations, at different historical moments, and in light of emerging challenges in human history. This Research Topic of Frontiers in Psychiatry is an opportunity for continued multidisciplinary reflection on these issues. In this editorial, we present a brief description of the articles that make up this volume.

Emotional exhaustion is a recognized element of the medical profession. The article titled “*A better way of life: The role of leisure activities on self-perceived health, perceived stress, confidence in stress management, and satisfaction with social support in psychiatrists and psychiatry trainees in Mexico*” analyzes the benefits of leisure activities for the wellbeing of a sample of psychiatrists and psychiatry residents (Lagunes-Córdoba et al.). The research team emphasizes the importance of promoting pleasurable activities among physicians in order to mitigate social determinants, such as work overload or burnout, which can undermine their mental health.

In accordance with the compromising effects that work responsibility and workload can exert on the wellbeing of healthcare workers, in the article titled “*Association of burnout with depression in pharmacists: a network analysis*”, the authors reflect on the similarities and differences between symptoms of depression and those of burnout in a group of frontline pharmacists in China (He et al.). It is established that emotional fatigue, depersonalization, and decreased work performance are dimensions of burnout related to depression, and therefore need to be considered for prevention.

Other publications in this Research Topic tackle neuropsychiatric pathologies of public health importance: psychosis and depression. The article titled “*Psychosocial factors associated with the risk of developing psychosis in a Mexican general population sample*” presents remarkable findings on how variables such as unhealthy family functioning, lower levels of academic achievement, having experienced either a natural disaster or the violent or unexpected death of someone close, a childhood history of abuse and neglect, and experiencing a higher degree of distress related to the COVID-19 pandemic are associated with a higher clinical risk of developing psychosis (Domínguez-Martínez et al.). In addition, the publication titled “*The duration of untreated psychosis among U.S. Latinxs and social and clinical correlates*” reports on the psychosocial factors involved in delays in seeking care among the Latino migrant population, with the element that stood out the most being the language barrier (Santos et al.). Both publications lead us to consider factors that should be taken into consideration for the development of prevention and treatment programs and policies, as well as in daily clinical practice, for earlier detection of psychosis and for the promotion of greater awareness of the approach to specific populations, such as migrants.

Depression is one of the most frequent and disabling psychopathologies. The article titled “*Social network typologies moderate the association of loneliness with depressive symptomatology in middle-aged and older adults*” addresses an interesting aspect of this condition, which relates to the influence of an individual's positive social networks in reducing the risk of depression or attenuating the impact of loneliness related to depressive symptoms (Shin and Park). The findings regarding the role of a diverse social network and family network suggest that it is important to complement the treatment of depression with interventions that improve the individual's prosocial skills, strengthening their membership in healthy social networks and decreasing the risk of interpersonal conflicts.

Finally, related to the issue of the family social network and its influence on predisposition to the development of eating disorders, the article titled “*Internalization of athletic body ideal as a mediating variable between family influence and body image of young women: a cross-cultural study of Polish, Italian, and Ukrainian women*” explores multicultural family groups to understand how family promotes the internalization of a body ideal, in which the aspiration for a slim and athletic body is not always accompanied by healthy habits and may be a risk for psychopathology (Izydorczyk et al.). The ideals of health and beauty are culturally variable; therefore, it is necessary to develop contextualized psychoeducation programs sensitive to sociocultural concepts, in order to promote healthy behaviors and decrease the risk of eating disorders.

As we can see, the point raised in the opening lines of this editorial is ratified in the findings and proposals presented by each of the contributions: in approaches to mental health, it is of vital importance to recognize the different elements of an individual's surrounding sociocultural environment in order to promote, maintain, and recover their mental health.

## Author contributions

All authors listed have made a substantial, direct, and intellectual contribution to the work and approved it for publication.
